# Potential health threats: the impact of hukou-based labour market discrimination on the health of rural migrants

**DOI:** 10.3389/fpubh.2025.1574960

**Published:** 2025-04-25

**Authors:** Qiuping Yi, Jingjing Li

**Affiliations:** ^1^College of Finance and Economics, Jimei University, Xiamen, China; ^2^School of Customs and Public Economics, Shanghai Customs University, Shanghai, China

**Keywords:** hukou system, labour market discrimination, rural migrants, health, Probit model

## Abstract

The hukou system is a population management policy in China. However, existing research has paid little attention to the relationships between hukou-based labour market discrimination and the health of rural migrants. To fill this research gap, we empirically examine whether and how hukou-based labour market discrimination impacts the health of rural migrants by employing an ordered probit model with the data from the 2017 China Migrants Dynamic Survey (CMDS). Our findings indicate that hukou-based labour market discrimination still prevails in Chinese cities. This discrimination negatively impacts rural migrants’ self-rated health. Then, addressing endogeneity and robustness issues using extended regression models and instrumental variables, the conclusions remain valid. We also found that “Whether to seek treatment for illness” emerges as a pivotal mechanism by which hukou-based labour market discrimination affects the health of rural migrants. Additionally, “enrollment in the New Rural Cooperative Medical Insurance” effectively mitigates the adverse effects of such discrimination on the health of rural migrants. Our findings underscore crucial guidance for advancing the reform of China’s hukou system and improving the health of rural migrants through targeted government policies and social initiatives.

## 1 Introduction

As a vital component of China’s industrial workforce, the rural migrants that have migrated to cities over the past several decades have made an indelible contribution to China’s economic growth. However, under the hukou system, the linkage of registration status with access to many urban public resources and services has prevented rural migrants from enjoying equal treatment as urban residents. Additionally, influenced by hukou-based labour market discrimination, these rural migrants face numerous implicit biases, including discrimination at legal, institutional, and social identity levels. This has prevented rural migrants from fully integrating into Chinese urban life, exacerbating the contradictions between urban and rural hukou migrants and leading to new socio-economic stratifications within cities (1). In response, the State Council of China issued guidelines in 2014 to further advance household registration system reforms, marking the official commencement of reforms aimed at dismantling policy-based discrimination. However, it is important to recognize that hukou-based labour market discrimination is likely to persist in the short term, and related social issues, such as the health of rural migrants, still not be swiftly resolved.

Existing literature widely recognizes the impact of the hukou system on employment, wages, housing satisfaction, savings rates, and subjective welfare among rural migrants. For instance, Huang et al. (2) demonstrated in their study on Chinese rural migrants that hukou system significantly influences rural workers’ access to non-agricultural employment. Liu and Kawata (3) used a developed version of the two-fold Blinder–Oaxaca decomposition based on our theoretical model, we find that the wage inequalities between permanent rural migrants and urban residents are caused by both the workers’ education and labour market tightness, as well as the father’s employment status, housing prices, and other factors. Fong et al. (4), utilizing the data from 2015 China Household Finance Survey, found that rural migrants’ homeownership significantly boosts housing satisfaction and overall life happiness. De Arcangelis and Joxhe (5), examining UK Household Longitudinal Study data from 1991 to 2008, explored migrant saving behaviors, treating savings and remittances as individual choices. Their estimates from a bivariate probability model indicated that short-term migrants save 26% more than permanent migrants. Additionally, studies like those by He et al. (6) analyzed data from based on 10,954 samples from the Chinese General Social Survey in 2013, 2015, and 2017, their results found that compared with having a rural hukou, having an urban hukou is correlated with higher levels of happiness in older adults. Moreover, those who had actively converted to an urban hukou or were born with it were happier, while passive conversion to an urban hukou was not significantly correlated with happiness. However, there has been limited focus on how hukou-based labour market discrimination affects the health of rural migrants. The socioeconomic disparities stemming from household registration not only led to economic inequalities (7, 8) but also significantly contribute to health inequalities (9), increasingly highlighting the health risks for rural hukou migrants. Thus, studying the impact of hukou-based labour market discrimination on the health of rural migrants holds significant relevance in Chinese high-quality development.

In response to the aforementioned research gaps, we utilized data from the 2017 China Migrants Dynamic Survey (CMDS) and employed an ordered probit model to empirically examine how hukou-based labour market discrimination impacts the health of rural migrants. Specifically, we applied the Oaxaca-Blinder decomposition technique to measure the extent of hukou-based labour market discrimination, which serves as the main explanatory variable in our study. We also used self-rated health data from the CMDS as the dependent variable. Our findings indicate that hukou-based labour market discrimination adversely affects the health of rural migrants. After conducting robustness checks using epidemiological data from the CMDS as an alternative to self-rated health and addressing endogeneity issues with instrumental variables, our results remained consistent. Furthermore, we discovered that the likelihood of seeking treatment for illnesses constitutes a critical pathway in the relationship between hukou-based labour market discrimination and the health of rural migrants; higher levels of hukou-based labour market discrimination reduce the likelihood that rural migrants will seek treatment when ill, thereby lowering their self-assessed health status. We also discussed the moderating role of the New Rural Cooperative Medical Scheme (NRCMS) from the perspective of economic displacement, finding that NRCMS helps mitigate the negative impacts of hukou-based labour market discrimination on the health of rural migrants.

This study makes two main contributions: First, it focuses on rural migrants in urban areas, examining the impact of hukou-based labour market discrimination on their health, incorporating insights from economics, sociology, and medicine, thereby augmenting the existing literature on the relationship between hukou system and resident health. Previous studies primarily explored the socio-economic impacts of hukou system from economic and sociological perspectives (10, 11), with less attention paid to the personal health effects among rural migrant groups. This research addresses this gap by emphasizing how hukou-based discrimination translates into tangible health disparities for rural migrants. Second, this research investigates the pathways through which hukou-based labour market discrimination affects rural migrants’ health and examines the moderating role of the NRCMS in this relationship. By doing so, it provides empirical evidence on whether the hukou system reforms have genuinely adopted a people-centered approach to health and well-being. In addition to policy measures such as the equalization of public services, this research highlights the importance of integrating the health of rural migrants into urban life—a key indicator of whether China’s development has truly achieved high-quality, inclusive progress. These contributions make a significant advancement in understanding the complex relationship between hukou-related labour market discrimination and migrant health, offering valuable insights for future policy reforms.

The remainder is structured as follows. Section 2 describes the literature review. Section 3 offers methodology, including the data, variables measured, and the empirical strategy. Section 4 analyzes and discusses the primary empirical findings, while Section 5 concludes.

## 2 Literature review

It is well known that the hukou system in China is tied to access to social welfare such as employment, housing, healthcare, education, and pensions (7, 9, 12–16). With the rise of a large-scale “floating population,” discrimination based on the hukou system has increasingly penetrated the labor market, leading to inequalities in sector entry, job acquisition, and income among different registration groups in cities (17). Classic sociological theories regard these aspects of inequality as crucial determinants of socioeconomic status (18). Consequently, mainstream perspectives acknowledge disparities in urban socio-economic status between groups, with rural migrants generally having a lower socio-economic status than their urban counterparts (19). This disparity fosters various prejudices, discrimination, and non-recognition toward rural migrants (10).

Numerous scholars have focused on hukou-based labour market discrimination, widely recognizing it as a critical tool for distinguishing worker identities and facilitating differential treatment in China’s labor market (20). This discrimination arises not only from the dual hukou system, which segments the labor market but also from the substantial changes that have occurred between urban and rural areas (21). Following China’s shift from a planned to a market economy, cities have experienced rapid development while rural areas have remained relatively underdeveloped, with the gap widening over time. Urban residents benefit from increasingly superior welfare linked to their hukou, and access to significantly greater social resources than their rural counterparts. This disparity has led to a natural sense of superiority among urban populations and prejudice against rural migrants.

Concurrently, rural migrants, recognizing their disadvantaged status, may settle for lower employment standards and wages. As hukou-based labour market discrimination deepens and becomes ingrained as a social custom, even urbanites who initially lacked prejudicial attitudes may feel compelled to conform to these norms, thus perpetuating the marginalization of rural migrants. Moreover, as this discrimination intensifies, it not only manifests in psychological biases but also extends into behavioral discrimination, leading to disparities such as unequal pay for equal work and occupational segregation in the labor market (22).

Additionally, discrimination fundamentally contradicts humanity’s eternal quest for fairness and equality and is detrimental to the long-term healthy development of society; thus, it has long been a focal issue in academic research. However, discriminatory practices are often difficult to capture and quantify in real life. Although some scholars have estimated the extent of hukou-based labour market discrimination by decomposing wage incomes (23–25), and others have explored the characteristics and extent of discrimination under occupational segregation (26), variations in specific issues and data sources among these studies have led to some heterogeneity in the calculated degrees of hukou-based labour market discrimination. Nevertheless, all studies unanimously provide evidence supporting the existence of hukou-based labour market discrimination, establishing it as a basic consensus among scholars regarding China’s urban labor market.

The hukou system is not only a unique product of China’s specific policy framework but also the essence of hukou-based labour market discrimination. The impact of such systems on national economic development and social health is not unique to China. Western scholars have long discussed the poorer health outcomes of lower socio-economic groups compared to their higher-status counterparts, a debate that has persisted for over half a century. Particularly since the release of the Black Report in 1980, health inequalities have become a significant topic of discussion among sociologists (27). Studies, including the Black Report and others from various countries, generally acknowledge a robust relationship between people’s socioeconomic status and their health (28–30). However, current theories and research have not definitively established causality, with ongoing debates between social causation—which argues that inequalities in social structure lead to differences in work environments, access to medical services, and health risks, thus better health among higher socio-economic groups (31, 32)—and health selection, which suggests that health status itself acts as a screening mechanism in status attainment, with healthier individuals achieving upward mobility. The focus of this study, however, is on the health impacts of hukou-based labour market discrimination, driven by exogenous policies, akin to how racial discrimination leads to socio-economic stratification in Western societies, such as findings by Cozier et al. (33) that increased discrimination correlates with higher hypertension rates among African American women in the U.S. labor market. Thus, this study argues that hukou-based labour market discrimination adversely affects the health of rural migrants.

The literature on China’s hukou system reveals significant socio-economic and health disparities between urban and rural populations. Studies show that rural migrants face barriers in accessing employment, higher wages, and public services due to their rural hukou status. This discrimination leads to broader socio-economic inequalities, including poor housing satisfaction and lower life satisfaction (34). While much of the research has focused on economic impacts, recent studies have begun to highlight the health disparities faced by rural migrants, which are exacerbated by limited access to urban healthcare services (35). The lack of access to affordable healthcare, coupled with poor living conditions and job insecurity, results in delayed treatment, untreated chronic conditions, and mental health issues (36). Public health insurance programs, such as the New Rural Cooperative Medical Scheme (NRCMS), have been shown to reduce healthcare barriers, yet these programs do not fully address the root causes of hukou-based discrimination (9).

In summary, we identify several key findings: First, hukou-based labour market discrimination indeed has numerous adverse effects on the work and lives of rural migrants; second, it negatively impacts the mental and physical health of rural migrants; and lastly, existing research on how this form of discrimination affects the health of rural migrants remains limited. Therefore, this paper examines the impact of labor market discrimination, driven by the hukou system, on the health of rural migrants and explores the mechanisms behind these effects. Additionally, it investigates whether the NRCMS, introduced by China’s Ministry of Health in 2003, can mitigate these impacts, aiming to provide empirical support for deepening reform of the hukou system in China.

To better understand the theoretical underpinnings of these health disparities, it is useful to conceptualize the various pathways through which hukou-based discrimination impacts health. The following framework illustrates how hukou-based labor market discrimination can lead to poor health outcomes among rural migrants. Specifically, it highlights how labor market exclusion and limited access to resources contribute to chronic stress, which in turn affects both mental and physical health. The framework also incorporates the role of the New Rural Cooperative Medical Scheme (NRCMS) as a moderating factor, which can alleviate some of these health challenges.

**Figure 1:**
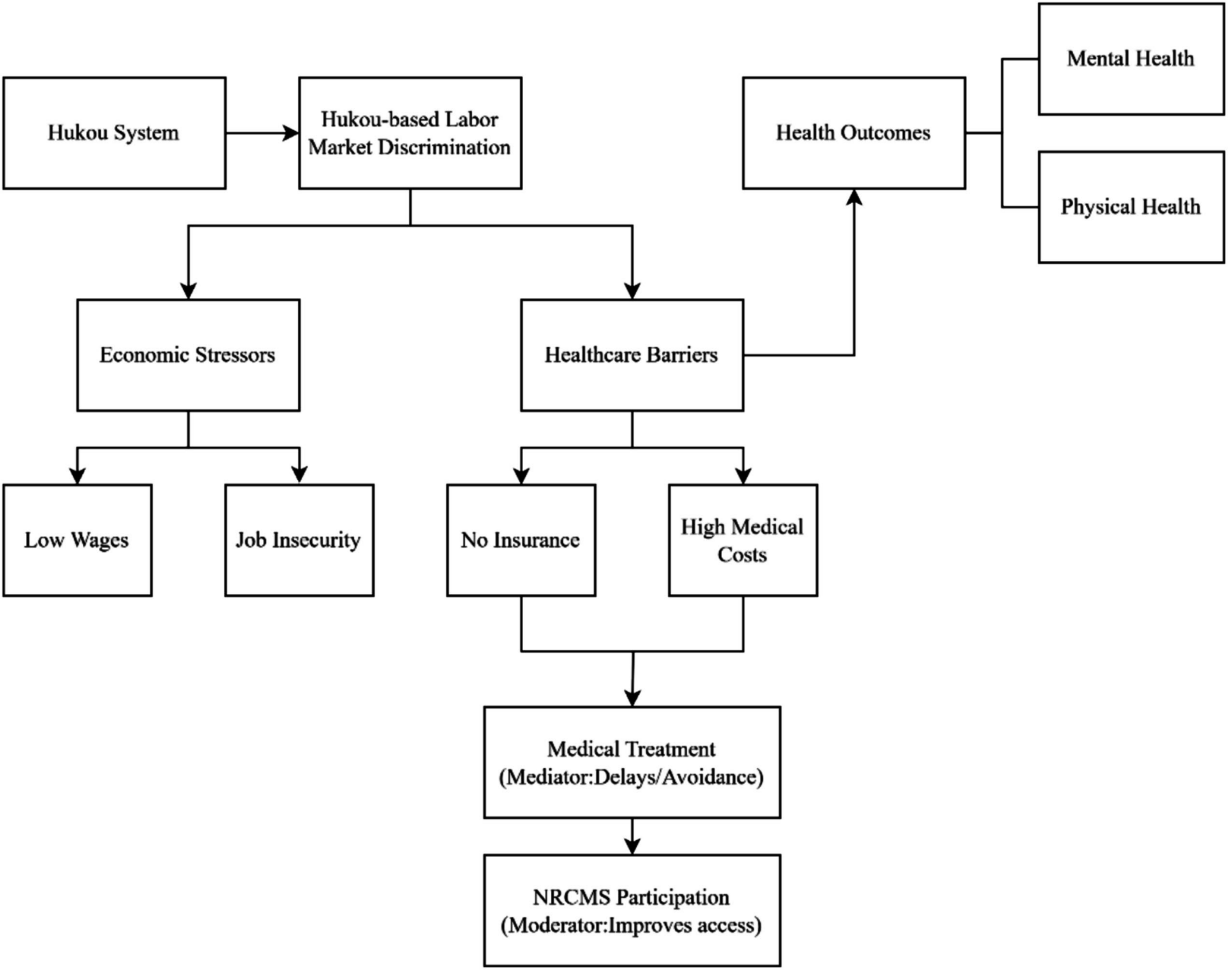
Theoretical framework.

Figure 1 presents the flowchart diagram that outlines the relationship between hukou-based labor market discrimination, socio-economic disparities, and health outcomes. This visual framework provides a comprehensive overview of the mechanisms we hypothesize to explain the health disparities faced by rural migrants. In the empirical section, we will test these hypotheses, focusing on the role of NRCMS in mitigating the adverse health impacts of hukou-based discrimination.

This framework not only serves as the basis for the hypotheses we propose but also positions this study within the broader literature on socio-economic stratification and health disparities among rural migrants in urban settings. The subsequent empirical analysis will test the validity of these theoretical pathways, contributing to the ongoing debate on the implications of the hukou system for migrant health. Hypotheses to be Tested.

Based on the theoretical framework outlined above, this study tests the following hypotheses to empirically examine the impact of hukou-based labor market discrimination on the health of rural migrants:

*H1*: Hukou-based labor market discrimination negatively affects the self-rated health of rural migrants.

*H2*: The likelihood of seeking treatment for illness acts as a critical mechanism linking hukou-based labor market discrimination and the self-rated health of rural migrants.

*H3*: Participation in the New Rural Cooperative Medical Scheme (NRCMS) moderates the negative effects of hukou-based labor market discrimination on the health of rural migrants.

## 3 Methodology

### 3.1 Baseline model

This study primarily examines the impact of hukou-based labour market discrimination on the health of rural migrants. We utilize data from the 2017 China Migrants Dynamic Survey (CMDS), specifically responses to the item “How is your health?” to measure the dependent variable in this study. A subjective item as the health metric is justified by representing a comprehensive and complex state, encompassing psychological and physical health conditions. This approach offers a significant advantage over most objective indicators, which tend to reflect only a narrow aspect of health. Moreover, since the dependent variable in this study is ordinal, we employ an ordered probit model, as outlined in Equation 1.

(1) $$Y_{i}=F\left(\partial dis_{i}+\beta X_{i}+\varepsilon_{i}\right)$$

where $Y_{i}$ represents the dependent variable, specifically self-assessed health. $dis_{i}$ is the key independent variable of interest, reflecting the degree of rural hukou discrimination at the urban level. $X_{i}$ includes control variables related to individual, family, urban, and provincial characteristics. F denotes a certain linear function, specified as in Equation 2:

(2) $$F\left({y_{i}}^{*}\right)=\{\begin{array}{c} 1{y_{i}}^{*}<\mu_{1}\;\\ 2\mu_{1}<{y_{i}}^{*}<\mu_{2}\\ \vdots \vdots \\ N{y_{i}}^{*}>\mu_{N-1}\; \end{array}$$

where $y^{*}$ represents the unobservable continuous variable underlying y .

### 3.2 Data description and statistical analysis

The CMDS includes extensive information on individual samples, such as family composition, income and expenses, employment status, mobility and residency intentions, health and public services, and issues related to social integration. Moreover, with a total sample size exceeding 140,000, the CMDS covers over 300 prefecture-level cities, states, and regions across China. Its widespread use in academic research, as documented by Chen and Wang (37), attests to its high credibility and persuasive power, making it suitable for the empirical analysis presented in this paper. Additional urban-level data used in this study were sourced from the statistical yearbooks of various cities for 2017.

Additionally, we undertook the following selection and processing of the data. Initially, given the focus of this paper on wage-based hukou discrimination against rural migrants in urban areas, we retained only those samples where individuals migrated for work or business purposes. Moreover, in calculating the degree of hukou discrimination at the city level, we noted a significant disparity in sample sizes between rural and urban migrants; on average, the sample size of rural migrants was approximately five times that of urban migrants. To ensure the accuracy of our discrimination measure, we excluded cities where the migrant population was less than 100. Furthermore, the original survey included an ordered response set for self-assessed health, ranging from healthy, basically healthy, unhealthy but independent, to unable to live independently. After cleaning the data according to these criteria and ensuring no missing values, only four samples remained in the “unable to live independently” category. For more precise results, we removed these samples, leaving the other three conditions as shown in Table 1. The results indicate that over 80% of the migrant population consider themselves healthy, with only a small fraction feeling healthy or unhealthy. The overall difference in health self-assessments between urban and rural migrants is minimal, although urban migrants’ percentage of self-reported health was still 1.58% higher than that of rural migrants.

**Table 1:** Statistics on self-assessed health.

	Items	Rural hukou migrants	Urban hukou migrants
Self-assessed health	Healthy	85.29%	86.87%
	Basically healthy	13.62%	12.53%
	Unhealthy but independent	1.09%	0.60%

Table 2 provides descriptive statistics of the sample’s characteristics and employment status. The results reveal that: (i) there is a significant wage disparity between urban and rural hukou migrants, with an average monthly wage difference of 1393.006 RMB. Specifically, rural hukou migrants earn an average monthly wage of 4150.422 RMB, compared to 5543.428 RMB for urban hukou migrants. (ii) There are substantial educational differences between the two groups. Education at or below junior high school, 68.53% of rural hukou migrants fall into this category, significantly higher than the 24.27% of urban hukou migrants. Conversely, a higher proportion of urban hukou migrants have received a high school/diploma level or higher. For instance, 27.06% of urban migrants have high school/diploma education compared to 20.58% of rural migrants, and the proportion of urban migrants with associate degrees or higher is approximately four times that of rural migrants. (iii) Regarding industrial distribution, both groups are predominantly employed in the tertiary sector, followed by the secondary, and then the primary sector. However, rural hukou migrants have a higher representation in the primary and secondary sectors than urban migrants, who are more concentrated in the tertiary sector. (iv) Regarding the employment sector, 17.86% of urban hukou migrants work in government, public institutions, and state-owned or state-controlled enterprises, a proportion significantly higher than that of rural hukou migrants. Urban hukou migrants are also less likely than rural ones to work in private enterprises and individual businesses. (v) Concerning occupational types, urban hukou migrants are significantly more likely to hold positions as managers or professional technicians, constituting about one-third of the urban migrant population, compared to only 7.32% of rural migrants. The proportions engaged in business activities are relatively similar between rural and urban migrants, at 25.65 and 20.57%, respectively. (vi) There are no notable disparities between the two groups for marital status, age, and gender distribution.

**Table 2:** Descriptive statistics of the migrant population.

Items		Rural hukou migrants	Urban hukou migrants
Average monthly wage (RMB)		4150.422	5543.428
Average monthly wage difference (RMB)		1393.006	
Marital status (married proportion)		81.94%	78.75%
Average age (year)		38.006	36.413
Gender (female proportion)		42.71%	43.99%
Education	Primary and below	19.26%	3.26%
	Junior high school	49.27%	21.01%
	High school/junior college	20.58%	27.06%
	Associate degree	7.47%	23.32%
	Bachelor’s Degree	3.16%	22.85%
	Graduate Degree	0.16%	2.49%
Industrial distribution	Primary sector	2.30%	1.32%
	Secondary sector	41.38%	34.56%
	Tertiary sector	56.32%	64.12%
Employment sector	Government, public institutions, and state-owned or state-controlled enterprises	5.08%	17.86%
	Private enterprises and individual proprietors	70.99%	63.49%
	others	23.93%	18.65%
Occupational types	Managers	0.24%	2.18%
	Professional technicians	7.08%	30.42%
	Business activities	25.65%	20.57%
	others	67.03%	46.43%

To sum up, our findings indicate a real wage gap between rural and urban hukou migrants. Besides the lack of significant differences in marital status, average age, and gender composition, the two groups exhibit notable disparities in education and employment, with rural migrants generally possessing lower educational levels and less stable, skilled employment. Thus, we argue that the wage disparities in the two groups are attributable to differences in education, employment, and the hukou system.

### 3.3 Hukou-based labour market discrimination measurement

Following the methods of Blinder (38) and Oaxaca (39), we applied the Oaxaca-Blinder decomposition technique to measure the degree of hukou-based labour market discrimination. The core principle of the Oaxaca-Blinder decomposition is to separate wage disparities into components attributed to individual characteristics and human capital differences, and those due to hukou-based labour market discrimination. After controlling for individual attributes such as age, education, the first time of leaving the household registration location (as indicated in the original survey), gender, marital status, industry, occupation, and the nature of the employing institution, we used the Oaxaca-Blinder decomposition to calculate the degree of hukou-based labour market discrimination across 51 cities. Our findings indicate that Zhengzhou exhibited the lowest level of this type of discrimination, at just 0.0101, suggesting that approximately 1% of the income disparity between urban and rural hukou migrants in Zhengzhou is due to labor market discrimination. Conversely, Guangzhou displayed the highest level, at 0.6784, meaning that 67.84% of the income differences between these groups in Guangzhou can be attributed to such discrimination. in addition, 38 cities had a discrimination level below 0.5, indicating that in 74.5% of the cities, less than half of the income disparity is caused by hukou-based labour market discrimination.

## 4 Results and discussions

### 4.1 Baseline results

Table 3 presents the baseline regression results on the impact of the health of rural migrants due to hukou-based labour market discrimination. First, the coefficient for hukou-based labour market discrimination is significantly negative as shown in column (1), which only includes core variables and individual-level control variables. This indicates a negative correlation between the hukou-based labour market discrimination and the self-rated health of rural migrants. Specifically, a 1% increase in hukou-based labour market discrimination leads to a 0.949% decrease in the health of rural migrants. Second, after incorporating city-level control variables, the results in column (2) still show a significantly negative coefficient. Here, each 1% increase in hukou-based labour market discrimination results in a 1.12% decrease in the self-rated health of rural migrants. Therefore, we conclude that hukou-based labour market discrimination significantly reduces the health of rural migrants.

**Table 3:** Baseline results.

Variables	(1)	(2)
	Self-assessed health of rural migrants	Self-assessed health of rural migrants
Hukou-based labour market discrimination	−0.949***	−1.120***
	(0.065)	(0.058)
Gender (female)	−0.097***	−0.165***
	(0.025)	(0.021)
Age (under 30)	1.655***	2.346***
	(0.103)	(0.058)
Age (between 30 and 60)	1.058***	1.606***
	(0.100)	(0.054)
Primary and below	−0.435***	−0.585***
	(0.030)	(0.025)
Bachelor’s degree or higher	0.030	0.073
	(0.071)	(0.062)
Married	−0.248***	−0.199***
	(0.043)	(0.035)
Family size of the immigrant area	0.081***	0.043***
	(0.013)	(0.010)
Migration distance	−0.157***	−0.172***
	(0.017)	(0.016)
Manager or technician	−0.013	0.128***
	(0.050)	(0.047)
Business	−0.026	0.096***
	(0.0297)	(0.028)
Secondary industry	0.088**	−0.215***
	(0.043)	(0.040)
Tertiary industry	0.096**	−0.007
	(0.040)	(0.040)
Government, public institutions, and state-owned or state-controlled enterprises	−0.025	0.038
	(0.049)	(0.047)
There are difficulties in their hometown	−0.544***	−0.538***
	(0.029)	(0.029)
There are difficulties before moving to the city	−0.355***	−0.372***
	(0.027)	(0.027)
Last month’s wage	0.031***	0.027***
	(0.004)	(0.004)
Per capita GDP of the immigrant city		−0.186***
		(0.055)
Population size of the immigrant city		0.035**
		(0.018)
Proportion of secondary industry in the immigrant city		−0.017***
		(0.004)
Proportion of tertiary industry in the immigrant city		−0.006*
		(0.003)
Province		−0.001*
		(0.001)
Constant cut1	−5.108***	−4.164***
	(0.128)	(0.354)
Constant cut2	−2.345***	−1.854***
	(0.123)	(0.354)
Observations	74,742	74,742
*R* ^2^	0.1120	0.1618

***, **, and * indicate significance at the 1, 5, and 10% levels, respectively; and standard errors are given in parentheses.

### 4.2 Robustness test

In our baseline regression, the dependent variable is self-rated health, which may be subject to biases stemming from personal subjective factors. To enhance the robustness of our baseline findings, we substitute self-rated health with specific illnesses. This analysis includes six common diseases and two chronic diseases. The common diseases, categorized as binary variables, are diarrhea, fever, rash, jaundice, conjunctival congestion, and the common cold. Similarly, the chronic diseases, also binary, encompass diabetes and hypertension.

Table 4 presents empirical results on the relationship between hukou-based labour market discrimination and the prevalence of six common diseases and two chronic conditions. Except for the fever, the prevalence of the other six common diseases correlates positively with the degree of hukou-based labour market discrimination. This suggests that hukou-based labour market discrimination decreases the health of rural migrants, consistent with the baseline results, thereby confirming the robustness and reliability of our findings.

**Table 4:** Robustness test.

Variables	Common diseases						Chronic diseases	
	Diarrhea	Fever	Rash	Jaundice	Conjunctivitis	Common Cold	Diabetes	Hypertension
Hukou-based labour market discrimination	0.306***	0.089	0.294**	1.308*	0.462***	0.136***	0.551*	0.367***
	(0.074)	(0.079)	(0.131)	(0.670)	(0.170)	(0.051)	(0.294)	(0.138)
Gender (female)	−0.144***	0.0979***	0.263***	0.001	−0.007	0.140***	−0.510***	−0.331***
	(0.027)	(0.028)	(0.047)	(0.221)	(0.062)	(0.018)	(0.110)	(0.052)
Age (under 30)	0.700***	0.234	−0.286	−0.251	−0.164	0.0563	−2.252***	−3.271***
	(0.183)	(0.167)	(0.250)	(1.040)	(0.347)	(0.104)	(0.336)	(0.158)
Age (between 30 and 60)	0.256	−0.171	−0.415*	−0.200	−0.128	−0.180*	−0.664**	−1.310***
	(0.182)	(0.166)	(0.247)	(1.019)	(0.343)	(0.103)	(0.286)	(0.129)
Primary and below	−0.002	0.101**	−0.008	0.631**	0.039	−0.009	0.822***	0.596***
	(0.038)	(0.040)	(0.065)	(0.246)	(0.082)	(0.025)	(0.107)	(0.053)
Bachelor’s degree or higher	0.129**	0.118*	0.137	−13.640	0.386***	0.120***	−1.555**	−0.565**
	(0.061)	(0.0651)	(0.106)	(615.900)	(0.136)	(0.046)	(0.717)	(0.227)
Married	−0.100**	−0.155***	−0.176**	−0.773**	−0.168*	−0.040	0.168	0.364***
	(0.041)	(0.044)	(0.073)	(0.322)	(0.098)	(0.029)	(0.203)	(0.103)
Family size of the immigrant area	0.045***	0.036**	0.022	0.140	0.060**	0.063***	−0.130**	−0.148***
	(0.013)	(0.014)	(0.024)	(0.099)	(0.030)	(0.009)	(0.053)	(0.026)
Migration distance	−0.017	0.011	−0.042	−0.089	0.062	0.089***	−0.019	−0.029
	(0.022)	(0.023)	(0.039)	(0.174)	(0.050)	(0.015)	(0.085)	(0.041)
Manager or technician	0.152***	0.139***	0.247***	−0.829	0.000	0.164***	0.374*	−0.146
	(0.047)	(0.050)	(0.080)	(0.725)	(0.115)	(0.035)	(0.222)	(0.122)
Business	−0.013	−0.109***	−0.040	0.184	−0.090	−0.102***	0.476***	0.204***
	(0.033)	(0.037)	(0.060)	(0.251)	(0.076)	(0.023)	(0.113)	(0.056)
Secondary industry	0.005	0.058	−0.090	−0.042	−0.117	0.170***	−0.204	−0.129
	(0.048)	(0.052)	(0.083)	(0.389)	(0.109)	(0.033)	(0.180)	(0.085)
Tertiary industry	−0.005	−0.015	−0.208***	−0.083	−0.039	0.063**	0.022	0.005
	(0.046)	(0.050)	(0.080)	(0.362)	(0.102)	(0.031)	(0.162)	(0.078)
Government, public institutions, and state-owned or state-controlled enterprises	0.146***	0.063	0.091	−0.899	0.055	0.052	0.088	0.209**
	(0.051)	(0.055)	(0.090)	(0.722)	(0.119)	(0.037)	(0.209)	(0.096)
There are difficulties in their hometown	0.433***	−0.009	0.003	0.052	0.026*	0.010***	−0.014	0.003
	(0.030)	(0.006)	(0.011)	(0.053)	(0.014)	(0.004)	(0.024)	(0.011)
There are difficulties before moving to the city	0.311***	−0.016***	−0.015	0.070	0.012	0.004	−0.004	−0.002
	(0.029)	(0.006)	(0.009)	(0.047)	(0.012)	(0.004)	(0.021)	(0.010)
Last month’s wage	0.00***	0.008*	0.001**	−0.002	0.001*	0.001***	−0.003	−0.002***
	(0.000)	(0.000)	(0.000)	(0.003)	(0.001)	(0.000)	(0.002)	(0.000)
Per capita GDP of the immigrant city	0.003***	0.004***	0.006***	−0.002	0.001	0.002***	0.002	0.002
	(0.001)	(0.001)	(0.001)	(0.006)	(0.001)	(0.000)	(0.002)	(0.001)
Population size of the immigrant city	0.005**	0.003	0.002***	−0.001***	0.000***	0.000***	0.000**	0.000***
	(0.002)	(0.002)	(0.000)	(0.000)	(0.000)	(0.000)	(0.000)	(0.000)
Proportion of secondary industry in the immigrant city	−0.007	0.001	0.013***	0.010	0.008***	0.015***	0.006	0.001
	(0.006)	(0.001)	(0.002)	(0.010)	(0.002)	(0.001)	(0.004)	(0.002)
Proportion of tertiary industry in the immigrant city	−0.015***	0.380***	0.534***	0.159	0.504***	0.389***	0.551***	0.402***
	(0.005)	(0.032)	(0.055)	(0.251)	(0.074)	(0.020)	(0.121)	(0.057)
Province	0.007***	0.270***	0.391***	0.0371	0.519***	0.287***	0.068	0.195***
	(0.001)	(0.031)	(0.051)	(0.245)	(0.068)	(0.021)	(0.110)	(0.053)
Constant	3.217***	2.471***	4.945***	12.070**	7.808***	2.669***	4.591**	2.521***
	(0.534)	(0.565)	(0.957)	(4.697)	(1.243)	(0.356)	(2.011)	(0.965)
Observations	56,094	56,459	57,039	57,103	57,109	56,214	56,194	56,194
*R* ^2^	0.0833	0.0652	0.0871	0.0792	0.0812	0.0884	0.0792	0.0882

***, **, and * indicate significance at the 1, 5, and 10% levels, respectively; and standard errors are given in parentheses.

### 4.3 Endogeneity concerns

**Figure 2:**
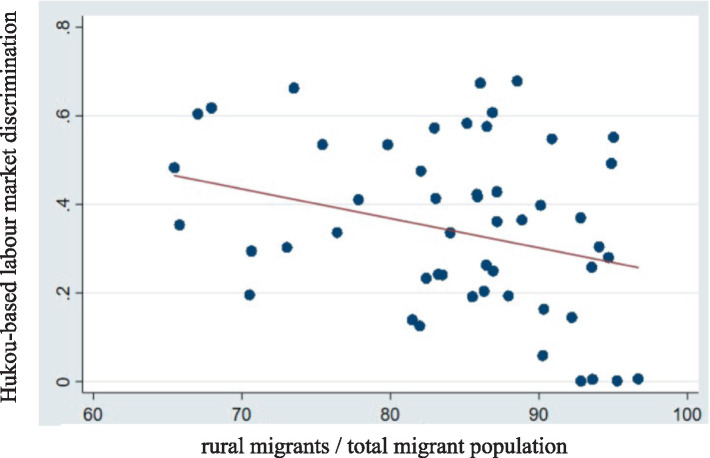
Trends in the hukou-based labor market discrimination and the proportion of rural migrants within the total migrant population.

The potential for endogeneity in the models discussed cannot be overlooked. Firstly, the study of the impact of hukou-based labor market discrimination on the health of rural migrants may involve self-selection issues. Specifically, rural migrants might anticipate the level of hukou-based labor market discrimination in a city before relocating and choose to move to cities with lower levels of discrimination based on their health. This implies that individuals make rational choices based on their health, which leads to potential endogeneity issues due to sample self-selection bias. Secondly, our study is constrained by the limitations of the original survey design and data availability, which might introduce endogeneity issues due to omitted variables and errors in the original data. To mitigate potential endogeneity issues, we employ an Extended Regression Model (ERM) to rerun the regression analysis. We introduce two instrumental variables: the proportion of rural migrants in the total migration population (labeled IV1) and the frequency of relevant terms in the 2017 government work reports (labeled IV2). The rationale for selecting these instrumental variables is as follows: First, a smaller proportion of rural migrants in the overall migration population is correlated with higher hukou-based discrimination, as the presence of a larger rural migrant population could lead to more awareness and less discrimination. Therefore, IV1 captures an exogenous variation in hukou discrimination based on the composition of migrants in a city. The second instrument is based on the frequency of terms related to hukou reform, migrant worker protections, and urbanization in local government work reports from 2017. These reports, delivered annually during local People’s Congress sessions, offer insights into the city’s commitment to addressing hukou-based discrimination. A higher frequency of these terms in government reports is hypothesized to correlate with reduced discrimination, as it suggests the city’s active engagement in reforming the hukou system. The rationale behind selecting these instruments is that both IV1 and IV2 are correlated with the level of hukou-based discrimination but are not directly related to the health outcomes of rural migrants. By using these instrumental variables, we aim to address potential endogeneity and improve the robustness of our estimates. This discrimination tends to increase as the proportion of rural migrants in the total migration population decreases (cf. Figure 2).

**Figure 3:**
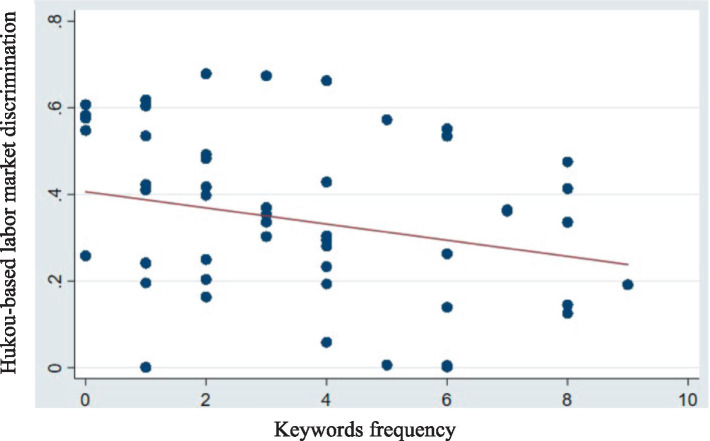
Trends in the hukou-based labor market discrimination and the keyword frequency related to urban hukou reform.

Additionally, the frequency of keywords relevant to research topics has become a common data source in recent academic studies (40, 41). This study focuses on hukou-based labor market discrimination, originally stemming from China’s dual hukou regulations. Despite reforms, the implicit discrimination linked to the hukou regulations does not disappear immediately; however, it is evident that easing these regulations effectively mitigates this form of discrimination. Thus, we employ the instrumental variable by the frequency of terms associated with hukou reform, protection of migrant worker wages, and the urbanization of the rural migrant population from the 2017 city government work reports. In China, local governments annually present these reports during sessions of the local People’s Congress and political consultative conferences, summarizing the past year’s activities and outlining the focus for the forthcoming year. The frequency of specific terms and phrases within these reports indicates the city’s attention to discrimination in the labor market based on hukou regulations. Thus, we manually collected the 2017 government work reports at the city level, focusing on keywords such as “hukou,” “migrant workers,” “rural migrant population,” “floating population,” “household registration,” and “urbanization.” We further analyzed the context of these keywords in each city’s government report to determine whether they conveyed intentions related to hukou reform, protecting the rights of migrant workers and the rural migrant population, and promoting their urbanization. If the context was relevant, we counted the frequency of these terms; if not, they were excluded. We posit that a higher frequency of these terms correlates with lower hukou-based labor market discrimination (cf. Figure 3).

Table 5 reports the empirical outcomes from the ERM incorporating the instrumental variables “the proportion of rural migrants in the total migration population” and “frequency of key terms in government work reports.” We found a significant negative correlation between two instrumental variables and hukou-based labor market discrimination, consistent with the results from the baseline regression analysis. Additionally, the *p*-value for Corr (e.dis, e.health) representing the correlation between these two residuals is significant and validates the appropriateness of our choice of instrumental variables. It also confirms that hukou-based labor market discrimination is an endogenous variable. However, in the adjusted model, there remains a significant negative correlation between hukou-based labor market discrimination and the self-assessed health of rural migrants, underscoring the robustness of our baseline results.

**Table 5:** Endogeneity test based on the ERM Model.

Variables	(1)	(2)
	Hukou-based labour market discrimination	Self-assessed health of rural migrants
Hukou-based labour market discrimination		−0.937***
		(0.110)
IV1	−0.007***	
	(0.001)	
IV2	−0.016***	
	(0.000)	
Gender (female)		−0.055***
		(0.014)
Age (under 30)		0.903***
		(0.062)
Age (between 30 and 60)		0.591***
		(0.060)
Primary and below		−0.261***
		(0.017)
Bachelor’s degree or higher		0.009
		(0.037)
Married		−0.113***
		(0.023)
Family size of the immigrant area		0.039***
		(0.007)
Migration distance		−0.061***
		(0.011)
Manager or technician		−0.019
		(0.027)
Business		−0.007
		(0.017)
Secondary industry		0.031
		(0.025)
Tertiary industry		0.027
		(0.023)
Government, public institutions, and state-owned or state-controlled enterprises		−0.036
		(0.027)
There are difficulties in their hometown		−0.283***
		(0.016)
There are difficulties before moving to the city		−0.212***
		(0.015)
Last month’s wage		0.014***
		(0.002)
Per capita GDP of the immigrant city		−0.001***
		(0.000)
Population size of the immigrant city		0.029***
		(0.010)
Proportion of secondary industry in the immigrant city		−0.010***
		(0.003)
Proportion of tertiary industry in the immigrant city		−0.002
		(0.003)
Province		−0.001*
		(0.001)
Constant	0.996***	
	(0.008)	
Corr (e.dis, e.health) p =0.002	0.063***	
	(0.021)	
Observations	74,742	74,742

***, **, and * indicate significance at the 1, 5, and 10% levels, respectively; and standard errors are given in parentheses.

### 4.4 Mechanism analysis

The next logical question to explore is: what potential channels warrant further investigation regarding the impacts on the health of rural migrants due to hukou-based labour market discrimination? In response to this, we delve deeper into two possible channels that may mediate or enhance this relationship: illness in the past year and whether it was treated.

The results in Columns (1) and (2) of Table 6 indicate that while hukou-based labour market discrimination significantly negatively affects the self-assessed health of rural migrants, its impact on their illness incidence over the past year is not significant. This suggests that the mechanism by which hukou-based labour market discrimination affects the health of rural migrants has not operated through their illness rates in the past year. Additionally, results in Column (3) demonstrate that hukou-based labour market discrimination significantly and negatively influences the illnesses that are treated, indicating that higher levels of such discrimination correspond to a lower likelihood of receiving medical treatment. Further, incorporating this form of discrimination and treatment status of rural migrants into a baseline model for a logit regression, the results in Column (4) reveal that the coefficient of hukou-based labour market discrimination is significantly negative, accounting for 4.11% of the total effect ^1^. This confirms that whether diseases are treated constitutes a crucial mechanism in the relationship between hukou-based labour market discrimination and the health of rural migrants: higher discrimination levels lead to a decreased likelihood of seeking treatment when ill, thereby decreasing their self-rated health level.

**Table 6:** Mechanism analysis.

Variables	(1)	(2)	(3)	(4)
	Illness in the past year	Self-assessed health of rural migrants	Illness was treated	Self-assessed health of rural migrants
Hukou-based labour market discrimination	0.031	−1.184***	−0.251***	−1.078***
	(0.051)	(0.070)	(0.085)	(0.070)
Gender (Female)	0.086***	−0.089***	0.027	−0.104***
	(0.018)	(0.026)	(0.031)	(0.026)
Age (under 30)	0.065	1.702***	−0.259	1.657***
	(0.104)	(0.109)	(0.200)	(0.107)
Age (between 30 and 60)	−0.163	1.069***	−0.105	1.073***
	(0.103)	(0.106)	(0.199)	(0.104)
Primary and below	−0.088***	−0.454***	0.153***	−0.431***
	(0.025)	(0.031)	(0.046)	(0.031)
Bachelor’s degree or higher	0.079*	0.014	−0.282***	0.010
	(0.045)	(0.072)	(0.067)	(0.072)
Married	−0.024	−0.241***	0.103**	−0.244***
	(0.029)	(0.045)	(0.049)	(0.045)
Family size of the immigrant area	0.055***	0.093***	−0.011	0.081***
	(0.009)	(0.014)	(0.016)	(0.013)
Migration distance	0.058***	−0.117***	−0.038	−0.123***
	(0.015)	(0.020)	(0.027)	(0.020)
Manager or technician	0.171***	−0.008	−0.050	−0.034
	(0.034)	(0.052)	(0.055)	(0.051)
Business	−0.151***	−0.038	0.047	−0.012
	(0.023)	(0.031)	(0.039)	(0.031)
Secondary industry	0.247***	0.106**	−0.044	0.068
	(0.033)	(0.046)	(0.059)	(0.045)
Tertiary industry	0.143***	0.076*	−0.172***	0.055
	(0.032)	(0.043)	(0.057)	(0.043)
Government, public institutions, and state-owned or state-controlled enterprises	0.007	−0.053	0.058	−0.060
	(0.036)	(0.051)	(0.062)	(0.051)
There are difficulties in their hometown	0.480***	−0.451***	−0.341***	−0.526***
	(0.020)	(0.030)	(0.035)	(0.030)
There are difficulties before moving to the city	0.233***	−0.342***	−0.010	−0.375***
	(0.020)	(0.028)	(0.034)	(0.028)
Last month’s wage	0.010***	0.030***	−0.010**	0.028***
	(0.003)	(0.004)	(0.004)	(0.004)
Per capita GDP of the immigrant city	0.010***	−0.000	−0.010***	−0.002***
	(0.000)	(0.001)	(0.001)	(0.001)
Population size of the immigrant city	0.000***	0.100***	−0.000***	0.004**
	(0.000)	(0.019)	(0.000)	(0.002)
Proportion of secondary industry in the immigrant city	0.022***	−0.017***	−0.008	−0.020***
	(0.004)	(0.006)	(0.008)	(0.006)
Proportion of tertiary industry in the immigrant city	0.005	−0.007	−0.009	−0.007
	(0.004)	(0.005)	(0.007)	(0.005)
Province	0.011***	0.001	0.005***	−0.001
	(0.001)	(0.001)	(0.001)	(0.001)
Injuries, illnesses, or physical discomfort in the past year		−0.775***		
		(0.026)		
Treatment for the injury, illness, or physical discomfort				0.183***
				(0.041)
Constant cut1	4.455***	−6.392***	−5.108***	−6.427***
	(0.359)	(0.495)	(0.681)	(0.493)
Constant cut2		−3.551***		−3.612***
		(0.494)		(0.492)
Observations	57,405	57,405	57,405	57,405
*R* ^2^	0.0830	0.1327	0.0974	0.1061

***, **, and * indicate significance at the 1, 5, and 10% levels, respectively; and standard errors are given in parentheses.

### 4.5 The moderating effect of the new rural cooperative medical insurance

In response to preventing rural poverty due to illness, China’s Ministry of Health introduced the new rural cooperative medical scheme (NRCMS) in 2003, aimed at reducing uncertainties in future medical expenses for rural residents. This system is now implemented nationwide and participated voluntarily. According to the CMDS2017 dataset, 75.58% of the rural migrants have enrolled in this insurance, 22.22% have not, and 2.2% are unsure of their enrollment status. We hypothesize that the implementation of this scheme may modulate the impact of hukou-based labour market discrimination on the health of rural migrants, as it reimburses a portion of large medical expenses, thereby alleviating the financial burden of illness on rural populations. Accordingly, we incorporated the “participation in NRCMS * hukou-based labour market discrimination” along with “participation in NRCMS” into our baseline regression model. Column (1) of Table 7 shows that the interaction term is positively significant at the 10% level, suggesting that participation in the NRCMS helps mitigate the negative impacts of hukou-based labour market discrimination on the health of rural migrants. Further, we validated the NRCMS moderating mechanism in whether diseases receive treatment. Using “disease treatment” as the dependent variable, the results in column (2) indicate that the interaction term “participation in NRCMS * degree of hukou-based labour market discrimination” is significantly positive at the 5% level, suggesting that participation in the NRCMS reduces the likelihood of untreated illnesses due to labor market discrimination based on household registration.

**Table 7:** The moderating effect of the new rural cooperative medical insurance.

Variables	(1)	(2)
	Self-assessed health of rural migrants	Treatment of illnesses among rural migrants
Hukou-based labour market discrimination	−0.845***	−0.788***
	(0.203)	(0.242)
Participation in the new rural cooperative medical insurance	−0.185***	0.139**
	(0.065)	(0.070)
Participation in the new rural cooperative medical insurance * Hukou-based labour market discrimination	0.244*	0.427**
	(0.135)	(0.180)
Gender (female)	−0.108***	0.026
	(0.026)	(0.032)
Age (under 30)	1.646***	−0.243
	(0.108)	(0.201)
Age (between 30 and 60)	1.049***	−0.094
	(0.105)	(0.199)
Primary and below	−0.430***	0.159***
	(0.031)	(0.047)
Bachelor’s degree or higher	−0.022	−0.268***
	(0.074)	(0.070)
Married	−0.261***	0.067
	(0.046)	(0.050)
Family size of the immigrant area	0.081***	−0.004
	(0.014)	(0.016)
Migration distance	−0.124***	−0.043
	(0.020)	(0.027)
Manager or technician	−0.045	−0.020
	(0.053)	(0.057)
Business	−0.006	0.047
	(0.031)	(0.040)
Secondary industry	0.066	−0.042
	(0.046)	(0.060)
Tertiary industry	0.040	−0.170***
	(0.043)	(0.057)
Government, public institutions, and state-owned or state-controlled enterprises	−0.075	0.055
	(0.052)	(0.064)
Proportion of secondary industry in the immigrant city	−0.017***	−0.010
	(0.006)	(0.008)
Proportion of tertiary industry in the immigrant city	−0.004	−0.010
	(0.005)	(0.007)
There are difficulties in their hometown	−0.534***	−0.338***
	(0.030)	(0.035)
There are difficulties before moving to the city	−0.377***	−0.019
	(0.028)	(0.035)
Last month’s wage	0.029***	−0.010**
	(0.004)	(0.004)
Per capita GDP of the immigrant city	−0.002***	−0.009***
	(0.001)	(0.001)
Population size of the immigrant city	0.041**	−0.000***
	(0.020)	(0.003)
Province	−0.001	0.005***
	(0.001)	(0.001)
Constant cut1	−6.592***	
	(0.501)	
Constant cut2	−3.785***	
	(0.499)	
Observations	55,888	55,888
*R* ^2^	0.1076	0.1305

***, **, and * indicate significance at the 1, 5, and 10% levels, respectively; and standard errors are given in parentheses.

## 5 Conclusion and policy implications

### 5.1 Main findings

This study empirically examines whether and how hukou-based labour market discrimination affects the health of rural migrants in Chinese cities. Specifically, it utilizes data from the 2017 CMDS and the Oaxaca-Blinder decomposition method, the study calculates the degree of hukou-based labour market discrimination across several Chinese cities. It then CMDS data combined with data from the China City Statistical Yearbook to empirically test the impact of this discrimination on the health of rural migrants. And further using extended regression models and alternative variables to address endogeneity and robustness issues. Additionally, the study explores the significant mechanisms by which hukou-based labour market discrimination affects the health of rural migrants and the moderating effect of the New Rural Cooperative Medical Insurance system. The study reaches the following significant conclusions: first, hukou-based labour market discrimination is still prevalent in Chinese cities, although the degree of discrimination varies by city. Therefore, hypothesis 1 is supported. Second, this discrimination negatively impacts rural migrants’ self-rated health, a result that holds true even after addressing endogeneity and conducting robustness checks. It follows that hypothesis 2 is valid. Finally, “whether to seek treatment for illness” is identified as a critical mechanism through which hukou-based labour market discrimination affects the health of rural migrants; furthermore, “participation in the New Rural Cooperative Medical Insurance” significantly mitigates the negative impact of this discrimination on their health. It proves that hypothesis 3 holds.

### 5.2 Policy implications

Based on our findings, we believe there is substantial room for advancing reforms in China’s hukou system. First, reforms should deepen by not only weakening and eliminating the dual hukou system at the institutional level but also by equalizing urban public services. This approach would fundamentally eradicate discrimination against rural hukous in urban labor markets. Second, the health conditions of rural migrants living in urban areas, who have remained marginalized in urban labor markets, warrant increased attention from both the government and society. Lastly, China needs to expand the coverage of the New Rural Cooperative Medical Insurance to reduce the healthcare burden on rural migrants, as our research indicates that this insurance scheme can alleviate the negative impacts of hukou-based labour market discrimination on the health of rural migrants.

To address the intertwined challenges of labor market discrimination and healthcare inequities, policymakers must adopt integrated reforms. First, enforce anti-discrimination laws to penalize hukou-based wage gaps and expand vocational training for rural migrants to access stable, higher-skilled jobs with health benefits. Simultaneously, integrate NRCMS with urban healthcare systems to enable seamless coverage and mandate employer premium contributions. Critical barriers to NRCMS—low enrollment, inadequate reimbursement, and interregional fragmentation—require mobile enrollment drives, tiered copayment subsidies, and a national digital platform for cross-province claims. Urban hospitals should be incentivized through performance-based funding to prioritize NRCMS patients. By linking labor policies and health reforms, China can dismantle systemic exclusion, ensuring rural migrants’ health reflects equitable rights, not hukou status.

## Data Availability

The raw data supporting the conclusions of this article will be made available by the authors, without undue reservation.

## Footnotes

^1^The mediated effect is calculated as −0.251 * 0.183, and the total effect is the coefficient for hukou-based labour market discrimination in the baseline model, which is −1.120. Using a similar method, the proportion of the mediated effect in the probit model is determined to be 2%.
